# Piggyback Add-on IOL as a Rescue Strategy after Incomplete Deployment of Primary IOL in an Excessively Small Capsular Bag and with Final Unexpected Refractive Benefit

**DOI:** 10.1055/a-2772-9011

**Published:** 2026-02-04

**Authors:** Giovanni Marco Conti, Nastasia Foa, Bao Khanh Tran

**Affiliations:** 1 General Ophthalmology Service, Hôpital ophtalmique universitaire Jules-Gonin, Lausanne, Switzerland; 2 General Ophthalmology Service, Centre dʼophtalmologie Y-Vision, Yverdon-Les-Bains, Switzerland

## Background

Cataract surgery in small eyes presents distinct challenges due to anatomical variations such as shallow anterior chambers and difficulty with lens calculation 1 . Several studies have found a generally positive linear correlation between capsular bag or lens diameter and axial length (AL); therefore, it is likely that small eyes are associated with smaller capsular bags 2 , 3 . It is widely acknowledged that capsular bag size plays a crucial role in determining postoperative IOL position and the likelihood of complications 3 . Therefore, precise IOL selection and meticulous surgical planning are essential. Moreover, small eyes are associated with a higher risk of intraoperative and postoperative complications such as endothelial trauma, iris prolapse, posterior capsule rupture, zonular rupture, malignant glaucoma and choroidal effusion as well as poorer refractive outcomes, when compared to eyes with normal axial length 1 . When refractive surprise happens, possible corrective strategies include IOL repositioning, IOL exchange, postoperative corneal refractive surgery, or piggyback IOL implantation 1 .

The aim of this article is to report a previously undescribed complication involving a rectangular IOL implanted in a small eye, highlighting the anatomical challenges associated with short axial length. This case underscores the importance of considering ocular anatomy when selecting and implanting IOLs, particularly in eyes with limited capsular space. Furthermore, it describes the use of a secondary piggyback add-on IOL as a rescue strategy following incomplete deployment of a primary IOL in a presumed undersized capsular bag, which resulted in an unexpected and clinically significant apparent accommodation. The article also explores potential optical mechanisms underlying this phenomenon.

## History and Signs

A healthy 64-year-old male presented with early-stage cataract, high hyperopia, and astigmatism (+ 7.25/−2.50 × 14°) in his right eye (RE), along with biometric findings consistent with a small eye: axial length (AL) of 20.04 mm, anterior chamber depth (ACD) of 2.75 mm, white-to-white (WTW) distance of 11.23 mm, and normal scleral thickness. Following preoperative counselling, we elected to proceed with cataract surgery targeting emmetropia. Given the patientʼs refraction state and axial length, the range of available intraocular lenses was limited. Finally, a custom-made rectangular monofocal toric IOL with four haptics (Soleko FIL611 T, diameter of 11.8 mm) was selected. We elected to use the IOLMaster 700 together with the Haigis formula for IOL power calculation.

**Fig. 1:**
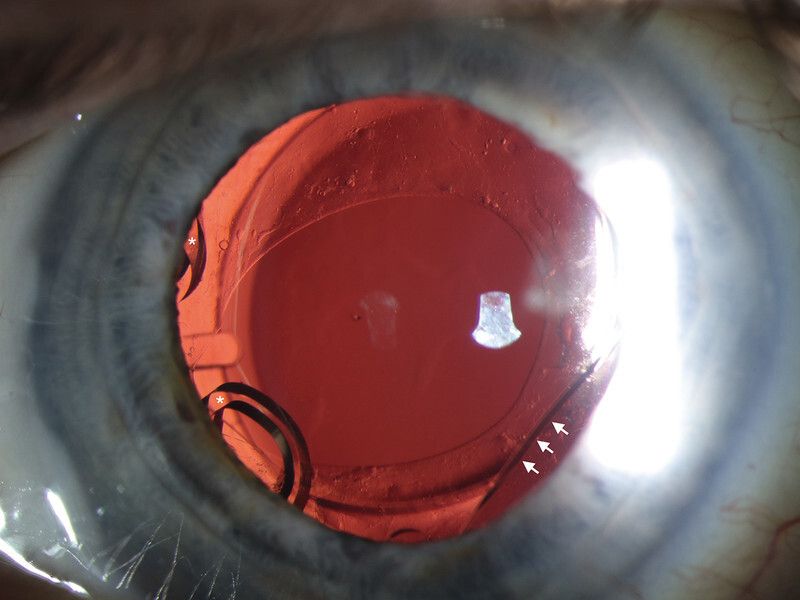
Slit-lamp photography showing two unfolded haptics of the primary IOL (white asterisk) and the piggyback IOL (white arrow), six months postoperatively (RE).

**Fig. 2:**
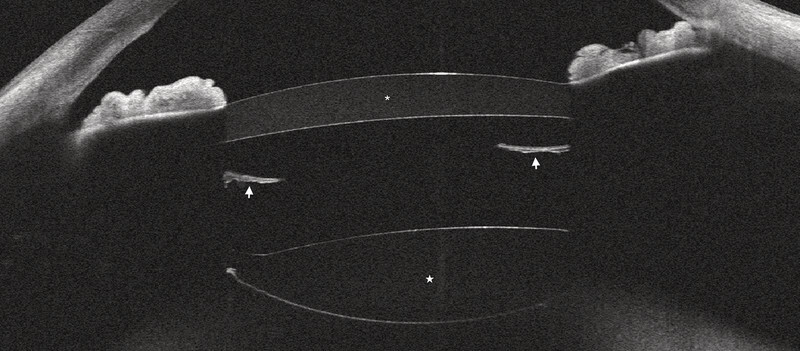
Anterior segment OCT showing the primary IOL (white star) with unfolded haptics (white arrow), the piggyback IOL (white asterisk) and narrowing of the nasal iridocorneal angle, six months postoperatively (RE).

The surgery was uneventful and intraocular lens was placed at the horizontal meridian in the capsular bag. The small pupil did not allow to visualize the border of the lens. Although the IOL was well-centered, postoperative uncorrected distance visual acuity (UDVA) was 0.5 logMAR (+ 2.75/−0.75 × 8°) at 10 days postoperatively without any significant ocular finding causing the hyperopic shift. Slit lamp examination showed improper deployment of the haptics both on temporal and nasal side ( Fig. 1 ). This postoperative hyperopic refractive shift was attributed to missdeployment of the haptics, resulting in posterior displacement of the IOL optic ( Figs. 1 and 2 ).

The patient was taken back to surgery 15 days after the first procedure to attempt capsular bag enlargement using viscoelastic and to reposition the IOL. Intraoperatively, the haptics remained improperly deployed, folded anteriorly toward the optic. The IOL failed to expand appropriately because its overall diameter was probably disproportionately large relative to the capsular bag dimensions. During manipulation, any attempt to rotate or reposition the IOL resulted in simultaneous movement of the entire capsular bag complex, indicating firm adhesion between the oversized IOL and the capsular equator. This mechanical coupling prevented safe rotation or extraction without risking zonular dehiscence or capsular rupture. Considering these intraoperative findings and the high risk of structural compromise, the surgeon elected to conclude the procedure, leaving the IOL in situ within the capsular bag.

## Therapy and Outcome

Benefits and disadvantages of corneal refractive surgery were discussed with the patient. After counselling, he declined the procedure because of concerns about nighttime glare and halos. As he frequently drove at night, he was particularly apprehensive about the risk of optical aberrations under mesopic conditions. Furthermore, given the high risk associated with IOL repositioning or exchange, and following discussion with both the patient and a refractive surgeon, the residual refractive error was corrected by implanting a secondary piggyback IOL in the sulcus.

The second procedure took place three months later and was uneventful ( Figs. 1 and 2 ). A 1stQ add-on IOL (1stQ, Germany) was implanted in the sulcus. Postoperative anterior chamber depth (ACD) was 2.4 mm. Anterior segment OCT revealed a well-opened iridocorneal angle in all quadrants, with a slightly narrower nasal angle observed in both miosis and mydriasis ( Fig. 2 ). Intraocular pressure (IOP) was 15 mmHg under both conditions and symmetric with that of the phakic left eye (LE). Slit-lamp examination showed no evidence of iris transillumination. Due to the nasal angle narrowing, a prophylactic laser iridotomy was performed.

**Fig. 3:**
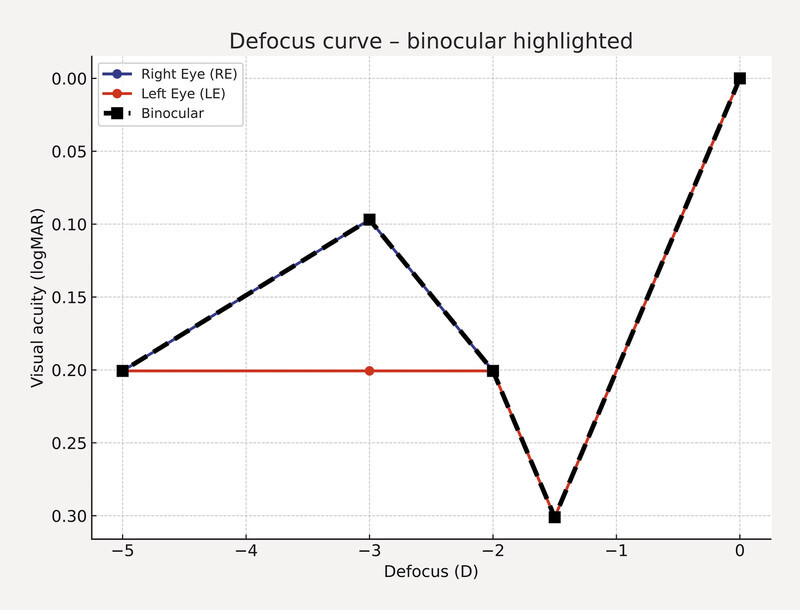
Defocus curve for the right eye (RE), left eye (LE), and binocular condition six months postoperatively. Visual acuity is expressed in logMAR as a function of defocus in diopters (D). The binocular curve is highlighted with a thicker dashed line, showing overall better tolerance to defocus compared with monocular performance.

**Fig. 4:**
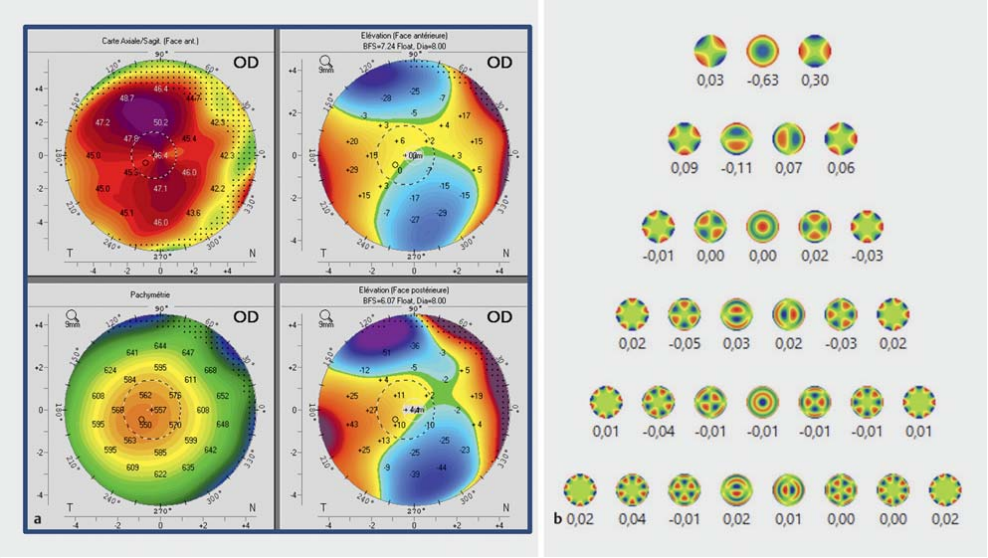
**a** Corneal topography showing with the rule astigmatism (RE). **b** Total aberrometry 3 Ø showing no spherical aberrations, horizontal coma of − 0.11 and vertical coma of 0.07 (RE) six months postoperatively.

The patient achieved a UDVA of 0.0 logMAR (1.0 decimal) in the right eye (RE) and, interestingly, reported good uncorrected near visual acuity (UNVA) of 0.1 logMAR at 33 cm ( Fig. 3 ). Final manifest refraction at six months postoperatively was − 0.25/−0.75 × 0° (objective refraction: − 0.25/−1.75 × 0°), the refraction was very similar at 1 months and 3 months postoperatively. This unexpected apparent accommodation was confirmed by defocus curve analysis and further investigated with total ocular aberrometry ( Figs. 3 and 4 ) at six months postoperatively, which revealed no spherical aberration (0 D), vertical coma of 0.07 D, and horizontal coma of − 0.11 D under photopic conditions (3 mm pupil diameter). Corneal topography demonstrated regular astigmatism. Pupil diameter measured 2.5 mm in photopic and 3.18 mm in mesopic conditions (measurements under scotopic conditions were not available).

In the fellow eye (LE), which had similar biometric parameters, a customized C-loop IOL (Soleko FIL622-1) with a maximum diameter of 15 mm was implanted. The IOL unfolded without complication. However, a similar postoperative hyperopic shift was observed. A sulcus-based piggyback 1stQ IOL was subsequently implanted. Final manifest and objective refractions were − 0.25/−1.25 × 10°, with a UDVA of 0.0 logMAR (1.0 decimal) and a UNVA of 0.2 logMAR (0.63 decimal) at 33 cm ( Fig. 3 ) at six months postoperatively. Findings in the LE were comparable to those observed in the RE with respect to corneal topography, aberrometry, and pupillometry.

Endothelial cell density (ECD) at six months postoperatively was 2,642 cells/mm² in the RE and 2,982 cells/mm² in the LE.

## Discussion

In this article, we describe a case of partial unfolding of a toric, monofocal, rectangular intraocular lens (IOL) with four haptics, and the surgical strategy adopted to manage this complication.

To our knowledge, the existing literature includes three reported cases in which one of the two haptics failed to unfold and one case in which both haptics unfolded only partially. All previously reported cases involved C-loop haptic IOLs. To the best of our knowledge, this is the first reported case of a rectangular, four-haptic IOL exhibiting such a complication 4 , 5 , 6 . In the most comparable previously published case (partial unfolding of two haptics), the underlying cause of the failure remained unclear 5 . Unfortunately, preoperative biometric and refractive data were not provided in that report. In the other two cases, incomplete unfolding was attributed either to a surgical error or to the presence of cortical remnants within the capsular bag 4 , 6 .

In the present case, we believe the complication resulted from a mismatch between the IOL diameter (11.8 mm) and an undersized capsular bag. This hypothesis is supported by multiple factors. The eye had a short axial length (20.04 mm), and several studies have demonstrated a positive linear correlation between axial length and capsular bag diameter 2 , 3 . Intraoperatively, despite multiple attempts and the use of viscoelastic to facilitate unfolding, the haptics failed to deploy completely. There were no residual lens fragments within the bag, and the haptics initially began to open but stopped upon contacting the anterior capsule. Finally, in the fellow eye, a C-loop IOL (15 mm diameter) was implanted without any unfolding issues, further supporting the likelihood of a size mismatch as the underlying cause.

While these considerations remain speculative due to the lack of preoperative lens diameter measurements, the surgical experience and biometric context strongly suggest an anatomical incompatibility between the IOL size and the capsular bag. The selection of an intraocular lens should always take into account the patientʼs individual ocular anatomy, particularly in eyes with smaller dimensions. In our case report, preoperative assessment of the capsular bag using ultrasound biomicroscopy or other measurement modalities might have aided in selecting a more appropriately sized IOL. C-loop IOL might have better chance to deploy in a small bag even if not completely with the optic in the right plane.

Following surgical evaluation and discussion with the patient, we adopted a surgical strategy based on the implantation of a piggyback add-on IOL. This approach is well documented in the literature as an effective solution for managing postoperative refractive surprises 1 . However, clinicians must remain aware of the potential complications in small eyes, including pupillary block or angle-closure glaucoma, iris chafing and pigment dispersion, which may lead to UGH syndrome and endothelial cell loss 7 .

After the second surgical procedure, the patient demonstrated an unexpected degree of apparent accommodation, achieving satisfactory uncorrected distance and near vision in both eyes. Apparent accommodation in pseudophakic eyes implanted with monofocal intraocular lenses refers to the ability to obtain functional vision at both distance and near despite the absence of true accommodation. This phenomenon is generally attributed to a combination of optical factors, including against-the-rule astigmatism, which increases depth of focus, and small pupil diameter, which enhances depth of field. Corneal multifocality and higher-order aberrations (HOAs), notably coma and trefoil, may further contribute, whereas axial movement of the IOL appears to have minimal influence 8 , 9 .

Small pupil diameter is a well-established determinant of increased depth of focus and is inversely correlated with pseudoaccommodation amplitude. Smaller pupils allow a broader range of clear vision across distances. In this case, the 2.5 mm pupil diameter likely played a significant role in the observed effect 9 .

Higher-order aberrations, particularly coma and trefoil, can extend the effective range of focus by generating multiple focal points, thereby compensating for the absence of true accommodation. A positive correlation between coma-like aberrations and apparent accommodation has been reported, particularly in eyes with with-the-rule astigmatism 10 . Although with-the-rule astigmatism is not as strongly associated with pseudoaccommodation as against-the-rule astigmatism, it may still modulate the distribution of corneal refractive power. However, recent evidence indicates that the magnitude of apparent accommodation does not differ significantly among eyes with varying astigmatic orientations, suggesting that the dominant contributing factors may shift depending on the individual corneal profile 10 .

In eyes with short axial length, the literature describes an increased depth of focus resulting from the combination of higher IOL power and the amplification of even minor IOL shifts. Although monofocal IOLs generally exhibit minimal movement, small displacements in short eyes can produce proportionally larger refractive changes, thereby enhancing pseudoaccommodation. Short axial length is also associated with increased corneal multifocality and HOAs, which further extend the range of functional near vision 11 . Smaller pupils and reduced spherical aberration may act synergistically to increase pseudoaccommodation in these eyes. However, the prevailing evidence suggests that in short pseudophakic eyes, the primary mechanism is optical amplification of static factors rather than dynamic accommodative change 11 .

The absence of negative spherical aberration and corneal multifocality in this case is notable, as both have been linked to enhanced pseudoaccommodation in other populations. Their absence supports the interpretation that the apparent accommodation observed here was primarily driven by small pupil size and higher-order aberrations, rather than by corneal multifocal effects 9 .

In the present case, both eyes exhibited with-the-rule astigmatism, a 2.5 mm pupil diameter, short axial length, and notable vertical and horizontal coma as well as trefoil aberration, but lacked negative spherical aberration and corneal multifocality ( Fig. 3 ). The similar outcome in the fellow eye, despite the absence of any perioperative complication and the use of a different main IOL, further supports the hypothesis that the apparent accommodation observed is predominantly related to intrinsic optical and anatomical characteristics rather than surgical factors. Given the ongoing controversies in the literature, the exact mechanism remains uncertain; however, it most likely results from the combined effects of small pupil size, short axial length, and significant higher-order aberrations, with a minor contribution from with-the-rule astigmatism.

Additionally, the patientʼs relatively young age may have played a role, as neuroplasticity could facilitate adaptation to multifocal-like vision. Similar effects have been reported in the literature in few cases; however, this remains an incidental benefit rather than a predictable or reproducible outcome. In fact, the predictability of this apparent accommodation is difficult to establish, as it likely depends on a combination of optical, anatomical, and neurological factors 8 .

Intraocular lens (IOL) selection should always take into account the patientʼs ocular anatomy, and in selected cases, additional preoperative imaging may be necessary to accurately assess capsular bag dimensions and ensure proper IOL sizing-particularly in small eyes, where a mismatch between the IOL and capsular bag size can lead to unexpected complications. In such situations, a secondary piggyback add-on IOL represents a safe and effective rescue option for correcting postoperative refractive errors, especially when corneal refractive surgery is contraindicated and the risk of IOL extraction or exchange is deemed too high.

In the present case, the sequential surgical approach successfully restored optimal visual function and, interestingly, provided an element of apparent accommodation, resulting in good uncorrected distance and near visual acuity. Although unpredictable, this outcome highlights how specific optical factors, including residual astigmatism, small pupil diameter, and higher-order aberrations, may, in rare instances, extend the depth of focus even in pseudophakic eyes implanted with monofocal IOLs.

The mechanism underlying apparent accommodation remains poorly understood and warrants further investigation. A deeper understanding of its optical and anatomical determinants could, in the future, allow surgeons to harness this phenomenon to improve functional outcomes. Overall, this case underscores the importance of individualized preoperative planning and flexible surgical decision-making to achieve optimal refractive and functional outcomes in challenging anatomical scenarios.

**Note**

This article was changed according to the Erratum on March 9, 2026.

**Erratum** In the above mentioned article the name of an author has been corrected. Correct is: Bao Khanh Tran.
