# Erratum: Piggyback Add-on IOL as a Rescue Strategy after Incomplete Deployment of Primary IOL in an Excessively Small Capsular Bag and with Final Unexpected Refractive Benefit

**DOI:** 10.1055/a-2826-2346

**Published:** 2026-03-09

**Authors:** Giovanni Marco Conti, Nastasia Foa, Bao Khanh Tran

**Affiliations:** 1 60541 General Ophthalmology Service, Hopital ophtalmique Jules-Gonin, Lausanne, Switzerland; 2 60541 General Ophthalmology Service,, Hôpital ophtalmique universitaire Jules-Gonin, Lausanne, Switzerland; 3 General Ophthalmology Service, Centre d’ophtalmologie Y-Vision, Yverdon-Les-Bains, Switzerland

 In the above mentioned article the name of an author has been corrected. Correct is: Bao Khanh Tran. This was corrected in online version on March 9, 2026.

